# Combination of fenretinide and ABT-263 induces apoptosis through NOXA for head and neck squamous cell carcinoma treatment

**DOI:** 10.1371/journal.pone.0219398

**Published:** 2019-07-05

**Authors:** Erin L. Britt, Sarina Raman, Kendall Leek, Casey H. Sheehy, Sung W. Kim, Hisashi Harada

**Affiliations:** Philips Institute for Oral Health Research, School of Dentistry, Massey Cancer Center, Virginia Commonwealth University, Richmond, Virginia, United States of America; Marshall University, UNITED STATES

## Abstract

The overall survival for recurrent or metastatic head and neck squamous cell carcinoma (HNSCC) remains low, with little progress made over decades. Cisplatin, most frequently used for HNSCC treatment, activates mitochondria-dependent apoptosis through the BCL-2 family proteins. We have previously demonstrated that the pro-apoptotic BH3-only protein, NOXA plays a critical role in this process. NOXA binds and inactivates anti-apoptotic MCL-1, while the BCL-2 inhibitor ABT-263 is capable of inactivating anti-apoptotic BCL-2 and BCL-X_L_. We hypothesized that combination of NOXA and ABT-263 treatment increases cell death by simultaneously inhibiting anti-apoptotic BCL-2 family proteins in HNSCC cells. Here, we demonstrated that combination of ectopic NOXA expression and ABT-263 enhanced apoptosis in p53-inactive, p53 wild-type, and human papillomavirus (HPV)-positive HNSCC cell lines. Furthermore, a retinoid derivative and an endoplasmic reticulum stress inducer, fenretinide, induced NOXA, and combination of fenretinide and ABT-263 strongly induced apoptosis in HNSCC cells regardless of the HPV or p53 statuses. We also found that MCL-1 and BCL-X_L_ are the primary targets of apoptosis induced by the combinations. These results will develop novel and alternative therapeutic strategies to directly modify the cell death machinery in HNSCC.

## Introduction

Head and neck cancer is the sixth leading cancer worldwide, with 600,000 cases annually; head and neck squamous cell carcinoma (HNSCC) accounts for more than 90% of these cases. Although multimodal treatment regimens for HNSCC, including surgery, chemotherapy, radiation, and immunotherapy have been developed, overall survival rates remain low over the past three decades [1, 2]. Induction chemotherapy with platinum-based compounds, taxanes, and 5-fluorouracil is beneficial for HNSCC patients, however, the prolonged use of these drugs is limited because of their toxicity and the eventual development of resistance. More recently, the combined use of molecularly targeted agents, such as EGFR-targeted cetuximab, with radiation has been proposed for management of patients with locally advanced HNSCC [3–5]. These types of therapies have shown promising results, but the survival of HNSCC patients has not changed dramatically. Innate or acquired resistance to chemotherapy is a major cause of treatment failure in cancer patients. As resistance to apoptosis is one fundamental mechanism that confers resistance [6, 7], one promising therapeutic approach is to utilize agents targeting molecular abnormalities that regulate resistance to apoptosis in HNSCC.

When cells undergo death triggered by chemotherapeutic agents, the BCL-2-family-dependent mitochondrial apoptotic pathway is activated. The BCL-2 family consists of three subgroups: pro-survival (e.g., BCL-2, BCL-X_L_, MCL-1), BH3-only pro-apoptotic (e.g., NOXA, BIM, BAD, BID), and multi-domain pro-apoptotic (e.g., BAX, BAK). Among these subgroups, BH3-only proteins cause cytochrome c release from the mitochondria by activating BAX and/or BAK, while the pro-survival proteins prevent this process [8–10]. However, p53, an initiator of this apoptosis pathway, is often mutated or deleted in many cancers including HNSCC, which causes them to be refractory to treatment [11, 12]. To combat such resistance, direct targeting drugs of the anti-apoptotic BCL-2 family has been recently developed. It has been reported that monotherapy with ABT-263 (navitoclax, targeted to inhibit BCL-2 and BCL-X_L_) [13] or ABT-199 (venetoclax, targeted to inhibit BCL-2) [14] is effective in chronic lymphocytic leukemia (CLL) and small cell lung cancer (SCLC) in clinical trials [15–17], and venetoclax has been approved by the FDA for *del*17p CLL patients [18, 19]. However, specific inhibitors of MCL-1 function are not yet in clinics (e.g. A-1210477, S63845 [20, 21]). Adding to the complexity of these approaches is the fact that tissue-specific interactions among the BCL-2 family members are observed in different tumor types. Although few studies have been performed in the context of the BCL-2 family in HNSCC, it has been proposed that MCL-1 expression might predict responsiveness to radiotherapy and chemotherapy in locally advanced HNSCC patients [22].

We have recently identified a BH3-only pro-apoptotic protein, NOXA, as a critical molecule that inactivates MCL-1 and induces cell death by cisplatin [23, 24], which is used as a standard therapy for locally advanced HNSCC. Since NOXA is an intrinsic and natural inhibitor of MCL-1, we ectopically expressed NOXA in HNSCC cells. Using a combination of NOXA and ABT-263 to inhibit all pro-survival proteins (i.e. MCL-1, BCL-X_L_, BCL-2), we observed efficient cell death in HNSCC cells. As an alternative NOXA inducer, fenretinide (N-4-hydroxyphenyl-retinamide), a synthetic retinoid derivative, was used in combination with ABT-263. It has been demonstrated that fenretinide induces NOXA by ER-stress in a p53-independent manner [25]. We found that the combination of fenretinide + ABT-263 efficiently induces cell death in HNSCC cells including p53-inactive, p53 wild-type, and human papilloma virus (HPV)-positive HNSCC cells.

## Materials and methods

### Cell lines and cell culture

HN8, HN12, and HN30 cells were provided by Andrew Yeudall (Augusta University). UMSCC1, UMSCC47, and UMSCC104 were provided by Yue Sun and Iain Morgan (Virginia Commonwealth University), respectively. These cells were cultured in Dulbecco’s Modified Eagle Medium (DMEM) (Life Technologies) and supplemented with 10% heat-inactivated fetal bovine serum (FBS) (Gemini Bio-Products, West Sacramento, CA) and 100 μg/mL penicillin G/streptomycin (Invitrogen) at 37°C in a humidified, 5% CO_2_ incubator. 293T cells were purchased from American Type Culture Collection (Manassas, VA).

### Virus production

NOXA-expressing adenovirus (Ad-NOXA) vector was constructed by inserting Flag-tagged NOXA cDNA (Ref) into pAdTrack-CMV vector (Addgene, Cambridge, MA), while the control adenovirus (Ad-Con) contained the vector alone. Adenoviruses were produced at the VCU shared resource core. Lentiviral short-hairpin RNA (shRNA) constructs were purchased from Sigma-Aldrich (St. Louis, MO). Constructs were transfected with psPAX2 and pMD2.G plasmids (Addgene, Cambridge, MA) into 293T cells with EndoFectin Lenti (GeneCopoeia, Rockville, MD), and the supernatants containing lentivirus were collected. The cell line of interest was infected with the lentiviruses and stable cell lines were established by puromycin (2 μg/ml) selection.

### Antibodies and chemicals

Fenretinide, ABT-263, and Q-VD-OPh were purchased from ApexBio Tech (Houston, TX). Antibodies for BIM, BAX, BAK, BCL-X_L_, Cleaved-PARP (Asp214), Cleaved-Caspase3, GAPDH (D16H11), HRP-linked anti-rabbit IgG, HRP-linked anti-mouse IgG, and Anti-Rabbit IgG conformation-specific antibodies were from Cell Signaling Technology (Beverly, CA); NOXA (114C307.1) was from Thermo Fisher Scientific (Waltham, MA); MCL-1 (ADI-AAP-240-F) was from Enzo Life Sciences (Farmingdale, NY); BCL-2 (100) was from Sigma-Aldrich. BAK antibodies for immunoprecipitation were purchased from Sigma-Aldrich (06–536 and AM03). ECL2 Western blotting substrate was purchased from Thermo Fisher Scientific (Rockland, IL). Annexin V-FITC and Annexin V-APC were purchased from Biolegend (San Diego, CA) and Thermo Fisher Scientific, respectively. Propidium Iodide was purchased from Sigma-Aldrich.

### Immunoprecipitation and Western blot analyses

Whole cell lysates were prepared with CHAPS lysis buffer [20 mM Tris (pH 7.4), 137 mM NaCl, 1 mM dithiothreitol (DTT), 1% CHAPS (3-[(3-Cholamidopropyl) dimethylammonio]-1-propanesulfonate)], 1:200 ratio of protease inhibitor cocktail, and 1:100 ratio of phosphatase inhibitor cocktails 2 and 3 (Sigma-Aldrich)]. For immunoprecipitation, equal amounts of protein were incubated with appropriate antibodies at 4°C overnight. Then, the antibody complexes were captured with protein A/G beads (Pierce, Rockford, IL) at 4°C for 1 hour. After washing three times with CHAPS lysis buffer, the beads were re-suspended in the same buffer + sample dyes and loaded onto a SDS-polyacrylamide gel. If the protein of interest was around 25 or 50 kD, the membrane was incubated with an anti-rabbit IgG conformation-specific antibody to prevent cross-reactive Ig heavy and light chains. For Western blots, equal amounts of proteins were loaded on a SDS-polyacrylamide gel, transferred to a nitrocellulose membrane, and analyzed by immunoblotting.

### Fluorescence Activated Cell Sorting (FACS) analyses

HNSCC cells were treated with Ad-NOXA, fenretinide (10 μM), ABT-263 (1 μM), or in combination. The control was treated with control adenovirus. Cells were trypsinized 24 hours later and re-suspended with 1 x Binding Buffer [10 mM Hepes (pH 7.4), 140 mM NaCl, and 2.5 mM CaCl_2_]. Then, Annexin-V and propidium iodide were added and flow cytometric analyses were performed using FACScan (BD Biosciences, San Jose, CA).

### Crystal violet assay

Cells were seeded in a 96-well plate with 1 x 10^4^ cells per well in 100 μL of medium. On the following day, cells were treated with combinations of Ad-NOXA or fenretinide with ABT-263. After 72 hours, cells were fixed with 10% Buffered formalin phosphate solution (Fisher Scientific, Hampton, NH) and then stained with 0.05% crystal violet gram stain (Fisher Scientific).

### Cell viability assays for Bliss Independence analysis

Cells were seeded in a 96-well plate with 1 x 10^4^ cells per well in 100 μL of medium. On the following day, cells were treated with various concentrations of fenretinide and ABT-263. After 72 hours, WST-1 assay (Sigma-Aldrich) was performed in duplicate to determine the cell viability. Drug synergy was determined using the Bliss independence analysis as previously described [26].

### Statistical analyses

Values represent the means ± S.D. for at least three independent experiments (N≧3). The significance of differences between the experimental variables was determined using the Student’s t-test. Values were considered statistically significant at P < 0.05.

## Results

### Ectopic NOXA expression alone induces apoptosis through BAK activation in HNSCC cells

**Fig 1:**
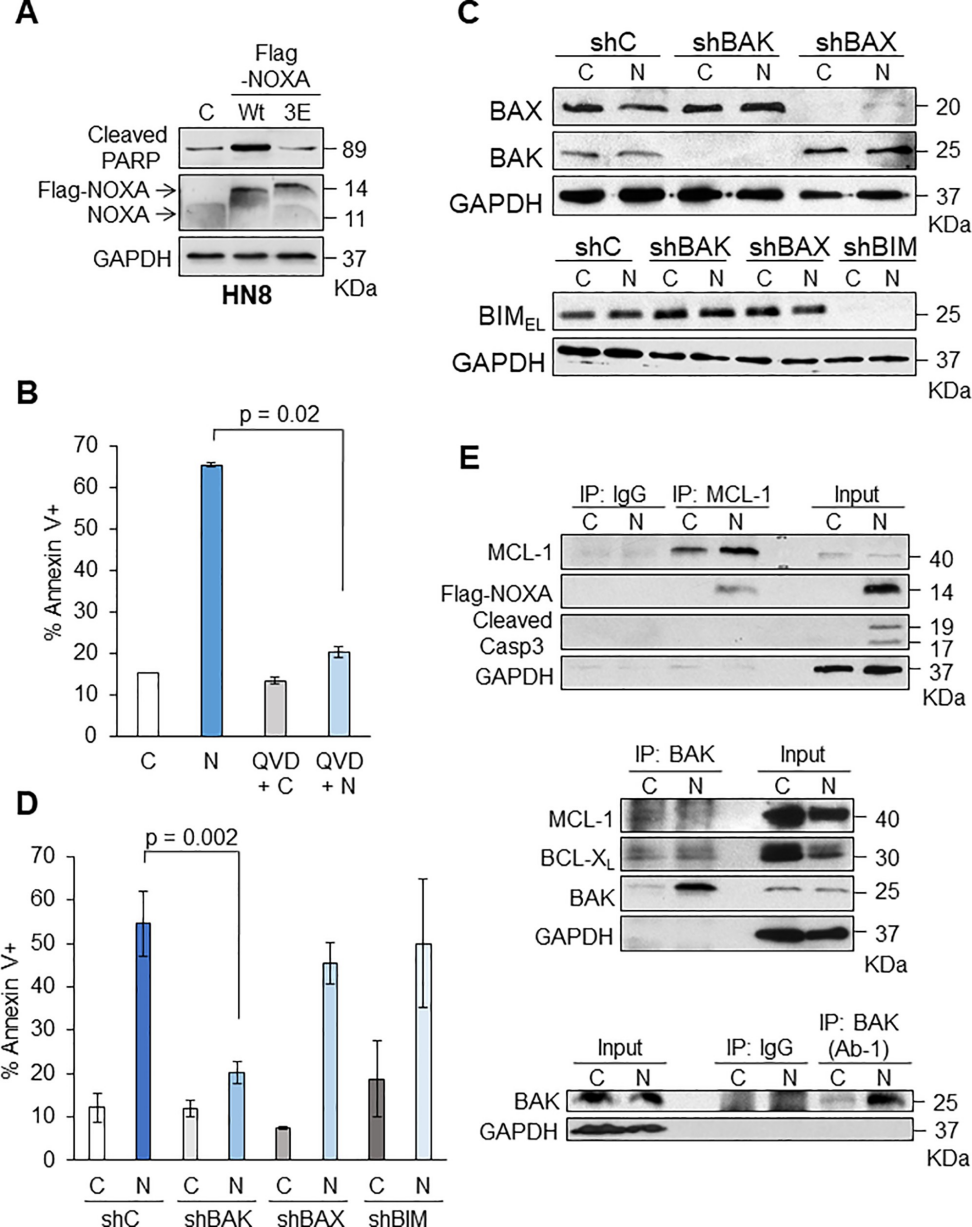
Ectopic NOXA expression alone induces apoptosis through BAK in HN8 cells. **(A)** HN8 cells were infected with lentivirus-encoding Flag-tagged NOXA wild-type (Wt), NOXA 3E or vector alone as control (C) for 24 h. Equal amounts of total extracts were subjected to Western blot analysis with the indicated antibodies. (B) NOXA alone induces apoptosis in HN8. Cells were treated with vector-control adenoviruses (Ad-Con, C), NOXA expressing adenoviruses (Ad-NOXA, N), Q-VD-OPH with Ad-Con (QVD+C), and Q-VD-OPH with Ad-NOXA (QVD+N). After 24 h, cells were analyzed with Annexin V-PI staining followed by FACS to determine the amount of apoptosis (N = 3). Values represent the means ± S.D. (C) BAK is mainly contributing to NOXA-induced cell death in HN8 cells. Lentiviruses encoding short-hairpin BAK, (shBAK), BAX (shBAX), BIM (shBIM), and non-targeting control (shC) were infected in HN8 cells and stable cell lines were established with puromycin selection. Cells were then treated with Ad-Con (C) and Ad-NOXA (N) for 16 h followed by Western blot analyses. (D) The cells in (C) were treated with Ad-Con and Ad-NOXA for 24 h followed by FACS analyses to determine total amount of apoptosis (N = 3). Values represent the means ± S.D. (E) NOXA binds to MCL-1 followed by BAK activation in HN8 cells. HN8 cells were treated with Ad-Con (C) and Ad-NOXA (N) for 16 h. Equal amounts of total extracts were incubated with IgG, anti-MCL-1 (top), anti-BAK (middle), or conformation-specific anti-BAK (bottom) antibodies. The input represents 20/500 of the immunoprecipitated lysates. **Top:** Immunoprecipitation with anti-MCL-1 followed by Western blots with the indicated antibodies. **Middle:** Immunoprecipitation with anti-BAK followed by Western blots with the indicated antibodies. **Bottom:** Immunoprecipitation with anti-BAK that detects a conformational change for BAK activation.

We have previously shown that cisplatin-induced cell death is largely mediated by NOXA in p53-inactive HN8 and HN12 HNSCC cell lines [24]. Thus, we examined whether NOXA overexpression could induce cell death in these cell lines. We first treated HN8 with the lentiviruses that expressing Flag-tagged NOXA [23]. NOXA overexpression alone efficiently induced cell death in HN8 cells (Fig 1A). NOXA 3E, in which the BH3 domain was mutated and could not bind to MCL-1 [27], did not induce cell death, suggesting the significance of NOXA/MCL-1 interaction (see also below). We confirmed that cell death induced by NOXA expression in HN8 cells was due to apoptosis, since cell death was mostly inhibited by a pan-caspase inhibitor, Q-VD-OPh (Fig 1B).

It has been demonstrated that NOXA specifically binds to and sequesters MCL-1 and then BAK is released from the BAK/MCL-1 complex to be activated [28]. We next addressed whether NOXA-induced apoptosis in HN8 cells solely depends on BAK. To examine this hypothesis, we introduced shRNAs specific for BAK, BAX, or BIM (Fig 1C). Once stable knockdown cells for each were established, we infected adenoviruses expressing Flag-tagged NOXA (Ad-NOXA) or control adenoviruses without NOXA cDNA (Ad-Con). Down-regulation of BAK mitigated NOXA-induced apoptosis, but not BAX or BIM (Fig 1D). After the introduction of NOXA in HN8 cells, a strong binding of NOXA to immunoprecipitated MCL-1 was detected (Fig 1E, upper panel). Then the interaction of BAK-MCL-1, but not BAK-BCL-X_L_, was decreased (Fig 1E, middle panel). Of note, the amount of immunoprecipitated BAK was much more after NOXA introduction, suggesting that NOXA was released from the complexes and became more accessible by the BAK antibodies. The conformational change of BAK was examined by using a conformation-specific antibody to immunoprecipitate the activated BAK. A clear increase for BAK conformational change was observed when NOXA was introduced, indicating BAK activation (Fig 1E, bottom panel). Taken together, these results indicate that NOXA-mediated BAK activation is sufficient to induce apoptosis in HN8 cells.

### Combination of NOXA expression and ABT-263 efficiently induces apoptosis in HNSCC cells

**Fig 2:**
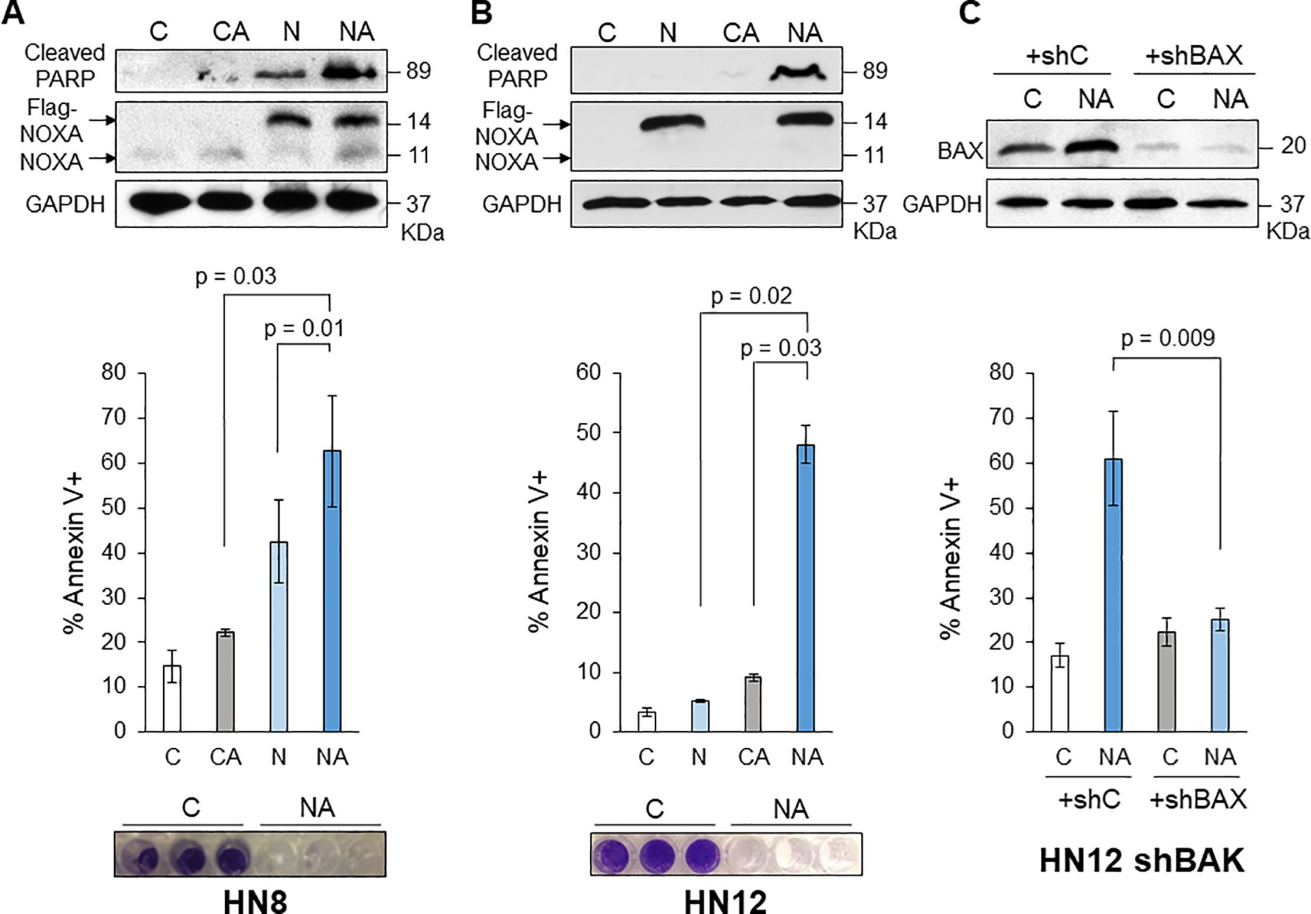
NOXA expression in combination with ABT-263 enhances cell death in HN12 cells. **(A) Top:** HN8 cells were treated with Ad-Con (C), Ad-NOXA (N) and/or ABT-263 (A) for 16 h and equal amounts of the total extracts were analyzed by Western blots with the indicated antibodies. **Middle:** Cells were treated with the same as above for 24 h and FACS analysis was performed to determine the amount of apoptosis (N = 3). Values represent the means ± S.D. **Bottom:** Cells were treated with the same as above for 72 h and stained with crystal violet. (B) HN12 cells were treated and analyzed as (A). Values represent the means ± S.D. for three independent experiments. (C) BAX and BAK are required for cell death induced by NOXA+ABT-263 in HN12 cells. **Top:** HN12 shBAK cells established in S1B Fig were infected with lentiviruses encoding shBAX or non-targeting control (shC) for 24 h. Cells were then treated with Ad-Con (C), Ad-NOXA (N) and/or ABT-263 (A) for 16 h and equal amounts of the total extracts were analyzed by Western blots with the indicated antibodies. **Middle:** Cells were treated with the same as above for 24 h and Annexin V-PI staining followed by FACS analysis was performed (N = 3). Values represent the means ± S.D.

Although NOXA expression alone efficiently induced cell death in HN8 cells, it did not induce cell death in HN12 cells. ABT-263 (navitoclax) has been developed as a BH3 mimetic that inhibits the function of BCL-2 and BCL-X_L_, but not MCL-1 [13]. Thus, we hypothesized that simultaneous inhibition of BCL-2 and BCL-X_L_ by ABT-263 and MCL-1 by NOXA efficiently induces apoptosis. Combined treatment of Ad-NOXA with ABT-263 appeared to have additive effects of cell death in HN8 cells (Fig 2A). In contrast, the combination synergistically induced cell death in HN12 cells, observed through the amount of cleaved PARP and Annexin V-positive population in FACS analysis (Fig 2B). The crystal violet staining showed that combinational treatment eliminated HN8 and HN12 cells in 72 hours (Fig 2A and 2B).

Ad-NOXA with ABT-263-induced cell death in HN12 cells is mainly apoptosis, because cell death was dramatically decreased when a pan-caspase inhibitor Q-VD-OPH was added with the combination treatment (S1A Fig). We next examined whether BAX and BAK are required for the combination-induced apoptosis in HN12 cells. To address this question, we introduced shRNAs specific for BAK, BAX, or BIM in HN12 cells. Once stable knockdown cells for each were established, cells were treated with Ad-NOXA and ABT-263 (S1B Fig). Interestingly, each knockdown did not significantly reduce the amount of apoptosis compared to the control. Thus, we transiently infected with the lentiviruses expressing shBAX into HN12 shBAK stable cells to generate the cells in which both BAX and BAK were down-regulated. Down-regulation of both BAX and BAK in HN12 cells mitigated cell death induced by combination (Fig 2C). These results suggest that both BAX and BAK are required for apoptosis induced by simultaneous inhibition of BCL-2, BCL-X_L_, and MCL-1 by ABT-263 and NOXA.

### Combination of NOXA induction by fenretinide and ABT-263 efficiently induce apoptosis in HNSCC cells regardless of the p53 status

**Fig 3:**
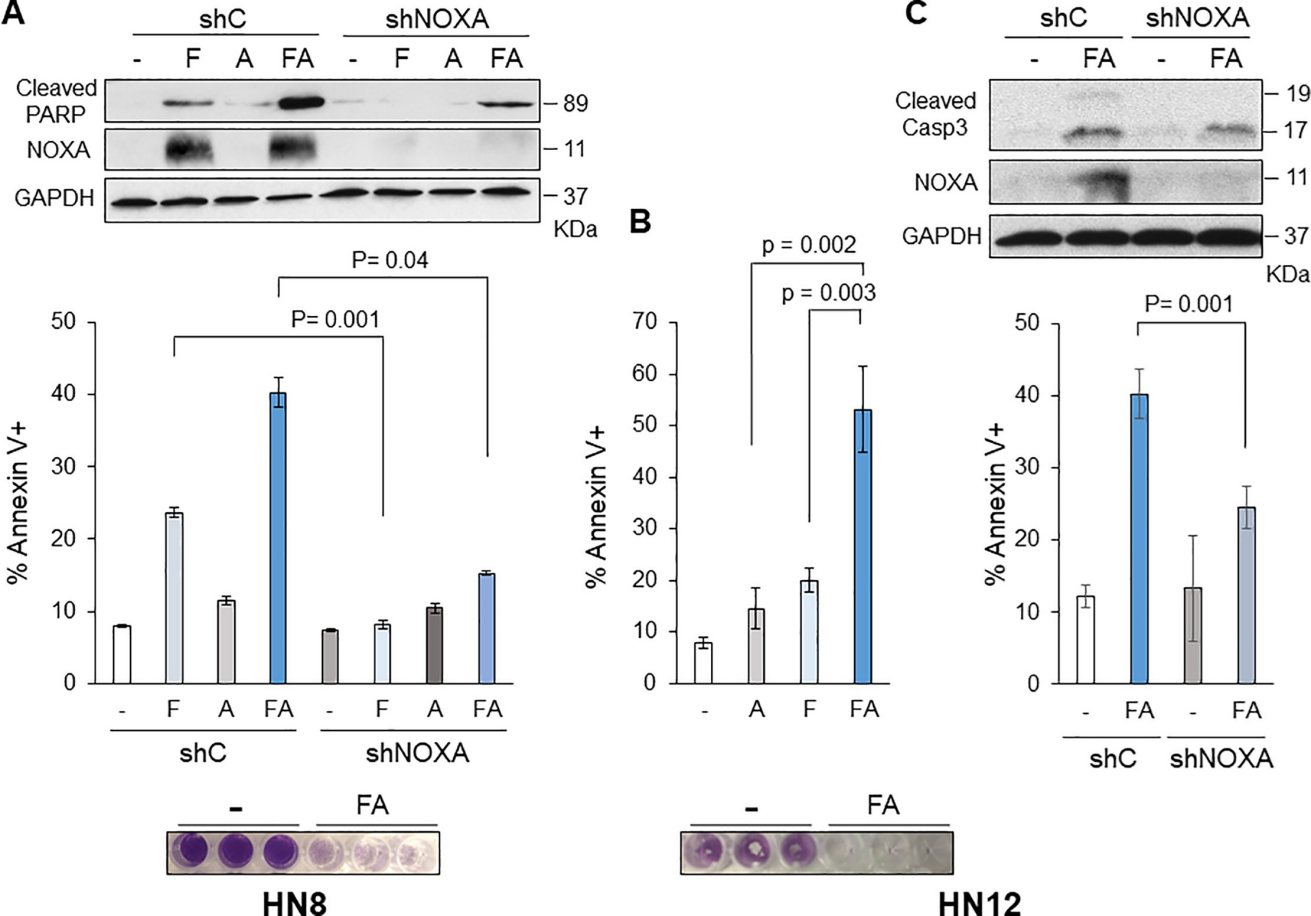
Combination of fenretinide and ABT-263 efficiently induces apoptosis in HN8 and HN12 cells. ***(A)* Top:** HN8 cells were infected with lentivirus-encoding shRNA for non-targeting control (shC) or NOXA (shNOXA). Cells were treated with fenretinide (F; 10 μM) and/or ABT-263 (A; 1 μM) for 16 h. Equal amounts of the total extracts were used for Western blot analysis with the indicated antibodies. **Middle:** The amount of apoptosis was determined by Annexin V-PI staining followed by FACS analyses (N = 3). Values represent the means ± S.D. **Bottom:** HN8 cells were treated with the same as above for 72 h and stained with crystal violet. (B) HN12 cells were treated and analyzed as (A). Values represent the means ± S.D. for five independent experiments. (C) **Top:** HN12 cells were infected with lentivirus-encoding shRNA for non-targeting control (shC) or NOXA (shNOXA). Cells were treated with fenretinide (F; 10 μM) and ABT-263 (A; 1 μM) for 16 h. Equal amounts of the total extracts were used for Western blot analysis with the indicated antibodies. **Bottom:** The amount of apoptosis was determined by Annexin V-PI staining followed by FACS analyses (N = 4). Values represent the means ± S.D.

We have recently demonstrated that ATF3/ATF4 transcription factors are critical mediators for cisplatin-induced NOXA in a p53-independent manner [24]. ATF3 and ATF4 are well known transcription factors activated by ER (endoplasmic reticulum)-stress. Fenretinide, a synthetic retinoid analogue and an ER-stress inducer, has been widely used in clinical trials for solid tumors treatment [29]. We have previously demonstrated that ATF3/ATF4 and NOXA are induced by fenretinide as similar level as cisplatin in HN8 cells [24]. Therefore, we hypothesized that the combination of ABT-263 and fenretinide could also induce efficient cell death in HNSCC, which would suggest that fenretinide could be an alternative NOXA inducer. Fenretinide strongly induced NOXA and modest cell death in HN8 cells (Fig 3A). When the lentiviruses encoding short-hairpin NOXA (shNOXA) were infected in HN8 cells, a clear decrease of fenretinide-induced cleaved-PARP and Annexin V^+^ population was observed as compared to HN8 shC (scrambled control) (Fig 3A). When fenretinide and ABT-263 were treated in combination, similar amount of NOXA was induced compared to fenretinide alone, and additive cell death was observed in HN8 cells. Combination-induced NOXA and cell death were also significantly reduced by shNOXA (Fig 3A). These results suggest that NOXA mainly contributes to cell death when HN8 is treated with fenretinide alone and in combination with ABT-263.

In contrast to HN8 cells, fenretinide treatment did not efficiently induce cell death in HN12 cells. However, combinational treatment of fenretinide with ABT-263 appeared to have synergistic effects in HN12 as compared to single treatment (Fig 3B). This combination-induced cell death was also significantly reduced by down-regulation of NOXA by shRNA (Fig 3C), suggesting NOXA contribution. Finally, the crystal violet staining showed that both HN8 and HN12 cells were completely killed with combinational treatment by the 72-hour point (Fig 3A and 3B), indicating the effectiveness of this combination regimen.

**Fig 4:**
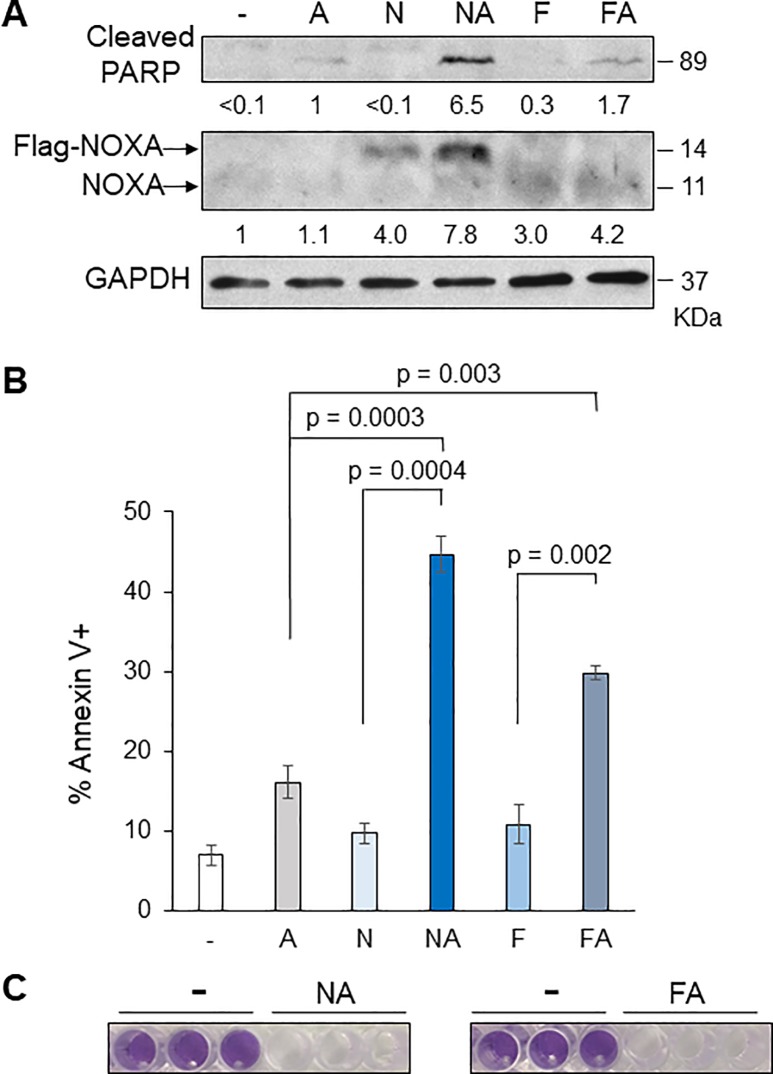
NOXA or fenretinide in combination with ABT-263 increases cell death in UMSCC1 cells. (A) UMSCC1 cells were treated with Ad-NOXA (N), fenretinide (F; 10 μM) and/or ABT-263 (A; 1 μM) for 16 h and equal amounts of the total extracts were analyzed by Western blots with the indicated antibodies. The relative amounts of cleaved-PARP and NOXA were determined by scanning densitometric analysis of the X-ray films using the NIH ImageJ program, normalized with GAPDH, and the relative value is shown at the bottom of the panel. (B) Cells were treated as above for 24 h and the amounts of apoptosis were determined by Annexin V-PI staining followed by FACS analyses (N = 3). Values represent the means ± S.D. (C) Cells were treated for 72 h and stained with crystal violet.

**Fig 5:**
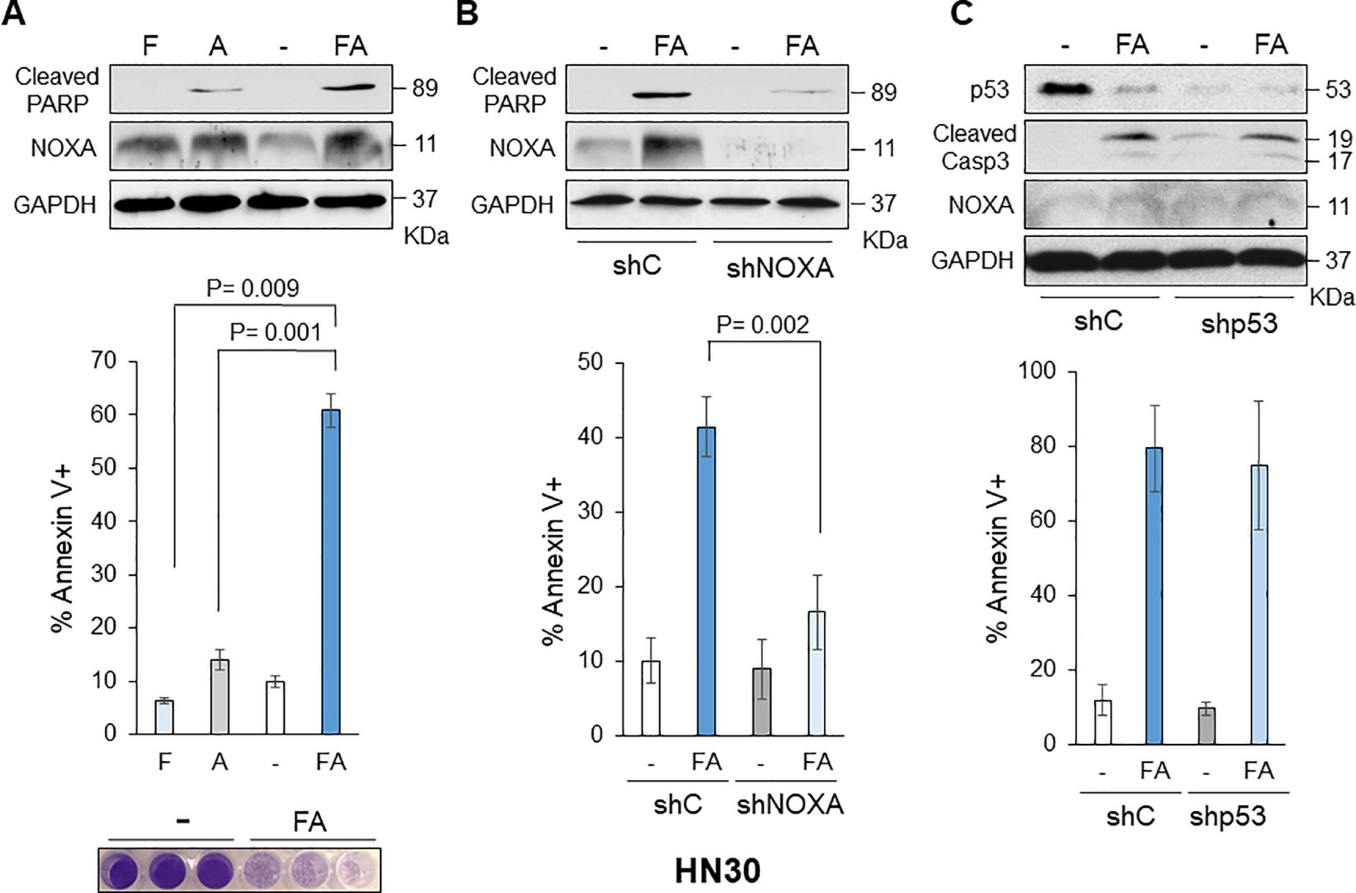
NOXA or fenretinide in combination with ABT-263 increases cell death in HN30 cells. (A) **Top:** HN30 cells were treated with fenretinide (10 μM) and/or ABT-263 (1 μM) for 16 h. Equal amounts of the total extracts were used for immunoblot analysis with the indicated antibodies. **Middle:** Cells were treated as above for 24 h and the amounts of apoptosis were determined by Annexin V-PI staining followed by FACS analyses (N = 4). Values represent the means ± S.D. **Bottom:** HN30 cells were treated for 72 h and stained with crystal violet. (B) HN30 cells were infected with lentivirus-encoding shRNA for non-targeting control (shC) or NOXA (shNOXA). Cells were then treated and analyzed as (A). Values represent the means ± S.D. for four independent experiments. (C) HN30 cells were infected with lentivirus-encoding shRNA for non-targeting control (shC) or p53 (shp53). Cells were then treated and analyzed as (A). Values represent the means ± S.D. for four independent experiments.

**Fig 6:**
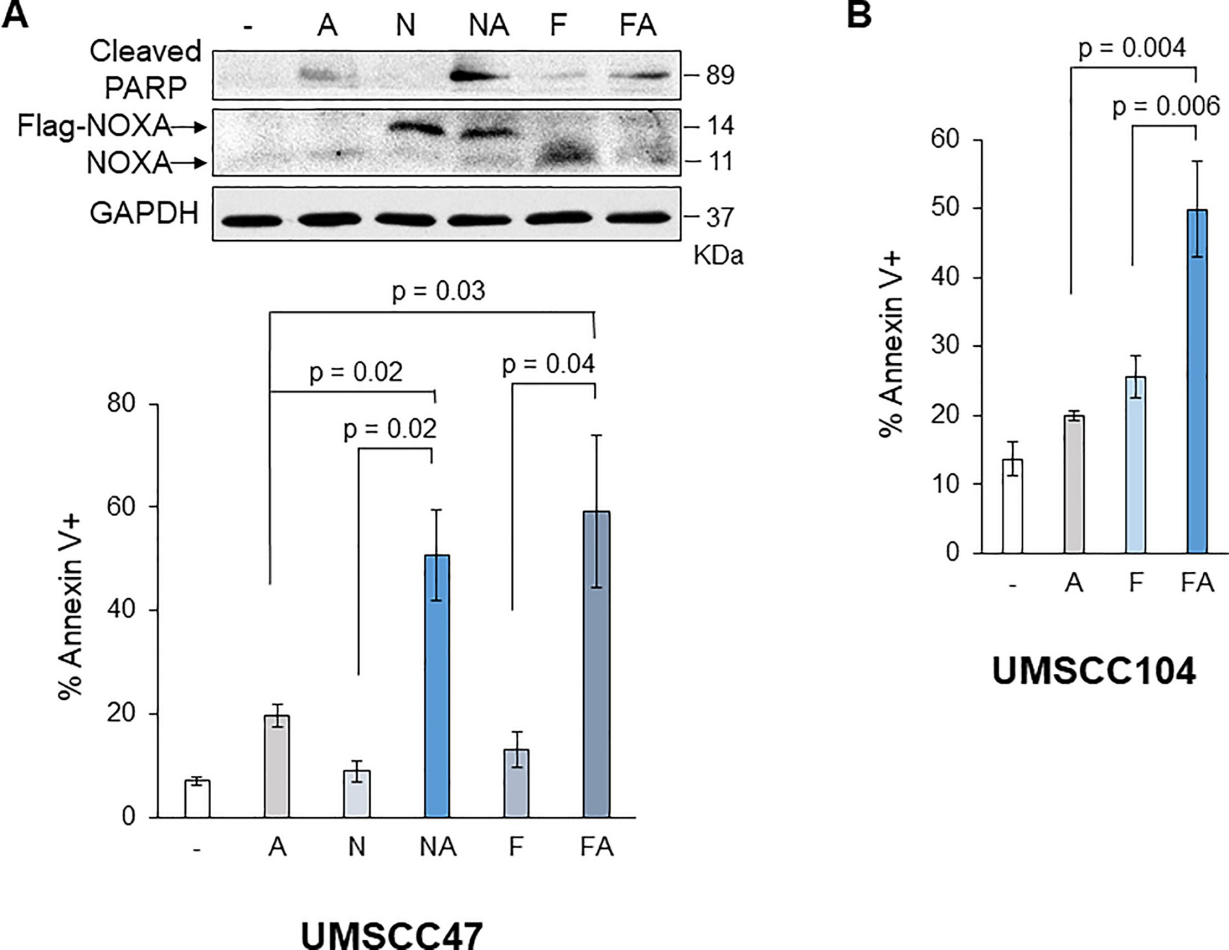
NOXA or fenretinide in combination with ABT-263 enhances cell death in HPV-positive UMSCC47 and UMSCC104 cells. **(A) Top:** UMSCC47 cells were treated with Ad-NOXA (N), fenretinide (F; 10 μM) and/or ABT-263 (A; 1 μM) for 16 h and equal amounts of the total extracts were analyzed by Western blots with the indicated antibodies. **Middle:** Cells were treated as above for 24 h and the amounts of apoptosis were determined by Annexin V-PI staining followed by FACS analyses (N = 3). Values represent the means ± S.D. (B) UMSCC104 cells were treated and analyzed as above. Values represent the means ± S.D. for four independent experiments.

NOXA was originally identified as a p53 target gene, however, fenretinide treatment induced NOXA in both HN8 and HN12 cells. HN8 has *p53* deletion and HN12 expresses a truncated form of p53 because a point mutation was found at the exon 7 splice donor site [30]. Thus, we further tested the cell lines that have different statuses of p53; HN30 and UMSCC1 possess wild-type p53 [30, 31], and UMSCC47 and UMSCC104 are HPV-positive, thus p53 function is inactivated [32]. We examined whether combinations of NOXA + ABT-263 and fenretinide + ABT-263 also efficiently induce cell death in UMSCC1 (Fig 4), HN30 (Fig 5A), UMSCC47 (Fig 6A), and UMSCC104 (Fig 6B). Bliss Independence analysis showed there was synergistic activity for the drug combination in each cell line (S2 Fig). Fenretinide alone slightly induced NOXA in UMSCC1 (Fig 4A), HN30, and UMSCC47, but could not induce cell death efficiently. However, the combination treatments could efficiently induce cell death in all cell lines tested, suggesting that fenretinide-induced NOXA and ABT-263 treatment increased cell death by simultaneously inhibiting the functions of anti-apoptotic BCL-2 family proteins and this strategy is effective regardless of the p53 status and function. Indeed, the interaction of BAK-MCL-1, BAK-BCL-X_L_ was significantly decreased after the treatment of fenretinide + ABT-263 (S3 Fig), suggesting that simultaneous inhibition of MCL-1 and BCL-X_L_ releases BAK (please also see Discussion). Furthermore, when NOXA was down-regulated by shRNA in HN30 cells, fenretinide + ABT-263-induced apoptosis was mitigated, indicating NOXA-dependent cell death (Fig 5B). In contrast, when p53 was down-regulated by shRNA in HN30 cells, fenretinide + ABT-263-induced NOXA and cell death were still induced to similar levels as control shRNA (Fig 5C), confirming that NOXA induction and cell death induced by fenretinide + ABT-263 is p53-independent.

### MCL-1 and BCL-X_L_ are the primary targets of apoptosis induced by NOXA+ABT-263 and fenretinide+ABT-263

**Fig 7:**
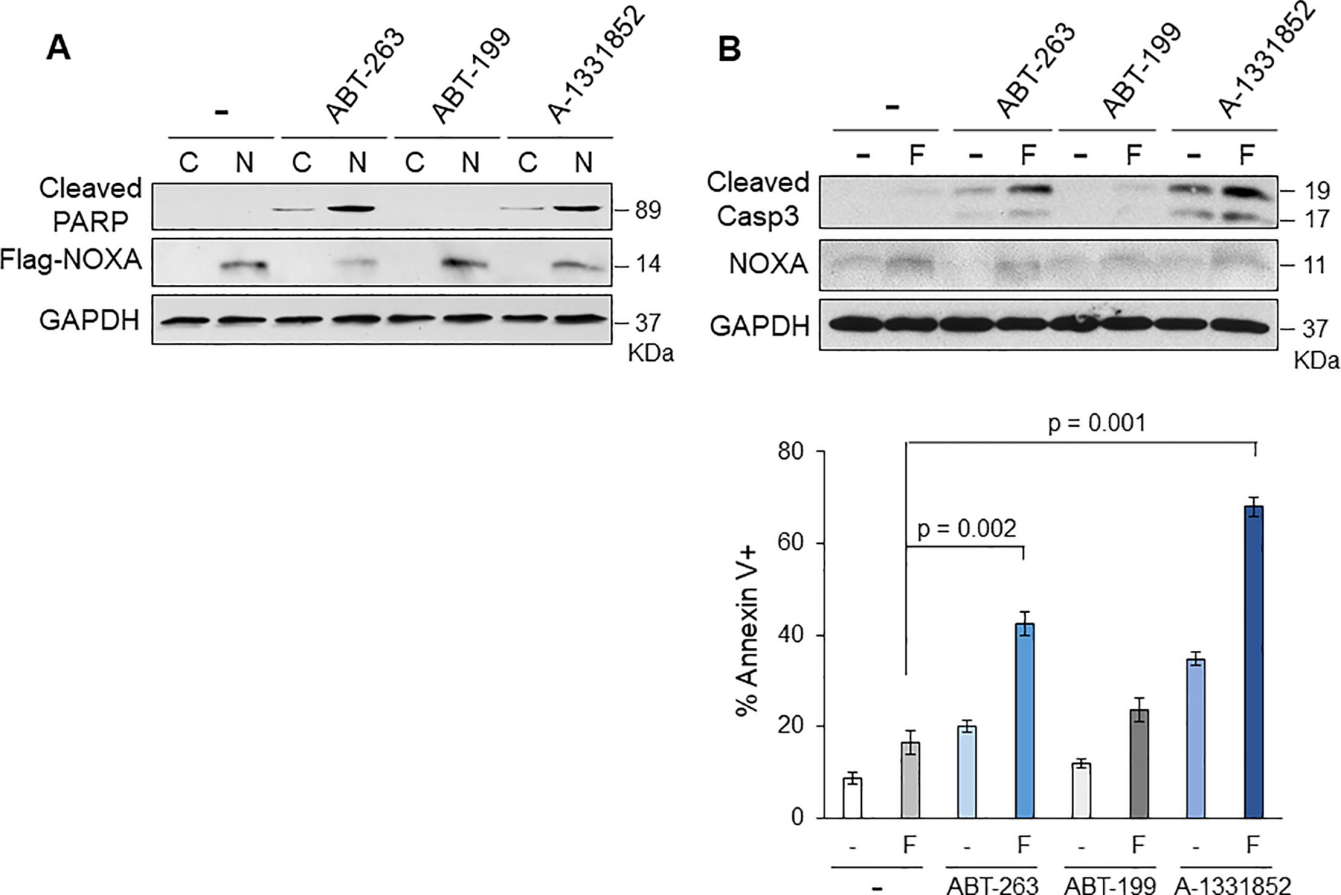
Inhibition of BCL-X_L_, but not BCL-2, is required for cell death induced by combined treatment with NOXA or fenretinide. **(A)** UMSCC1 cells were treated with Ad-Con (C), Ad-Noxa (N) and ABT-263(1 μM), ABT-199 (1 μM), or A-1331852 (1 μM) for 24 h. Equal amounts of total extracts were subjected to immunoblot analysis using the indicated antibodies. **(B) Top:** UMSCC1 cells were treated with fenretinide (F) and/or ABT-263, ABT-199, or A-1331852 for 16 h. **Bottom:** UMSCC1 cells were treated with fenretinide (F) and/or ABT-263, ABT-199, or A-1331852 for 24 h and the amounts of apoptosis were determined by Annexin V-PI staining followed by FACS analyses (N = 4). Values represent the means ± S.D.

ABT-263 binds and inhibits both BCL-2 and BCL-X_L_ [13]. We next addressed whether BCL-2 or BCL-X_L_ inhibition is required for combination-induced apoptosis. To this end, we used ABT-199 (venetoclax, a BCL-2 specific inhibitor) [14] and A-1331852 (a BCL-X_L_ specific inhibitor) [33] to determine the specificity. The result clearly showed that A-1331852 + Ad-NOXA combination induced apoptosis as similar as ABT-263 + NOXA combination, but BCL-2 inhibition by ABT-199 did not show apoptosis (Fig 7A). When cells were treated with fenretinide + A-1331852, similar amount of cell death was observed as fenretinide + ABT-263, but not fenretinide + ABT-199 (Fig 7B). These data strongly suggest that MCL-1 and BCL-X_L_ are the primary pro-survival proteins in HNSCC and efficient cell death can be induced when both proteins are simultaneously inhibited.

## Discussion

We have previously demonstrated that cisplatin induces apoptosis in p53-inactive HNSCC cells and the ATF3/ATF4-NOXA pathway is critical for this effect. Since NOXA is capable of binding and inhibiting the anti-apoptotic protein MCL-1, we hypothesized whether (1) NOXA expression alone could induce cell death in HNSCC cells; (2) a combination of NOXA expression and the BCL-2/BCL-X_L_ inhibitor ABT-263 could synergistically induce cell death; (3) a retinoid analogue fenretinide that induces the ATF3/ATF4-NOXA pathway could be an alternative for NOXA expression and the combination with ABT-263 efficiently induces cell death. Ectopic NOXA expression alone induces apoptosis in HN8 cells (Fig 1). In contrast, NOXA expression alone did not induce significant amounts of cell death in other cell lines examined (Figs 2, 4 and 6). The results from gene knockdowns and immunoprecipitations (Fig 1C–1E) indicate that activation of BAK by NOXA that sequesters MCL-1 is sufficient to induce efficient apoptosis at least in HN8 cells. Consistently, fenretinide alone induced significant levels of NOXA and cell death in HN8 cells (Fig 3A), but not in other cells tested (Figs 3B, 4, 5A and 6). In contrast, we demonstrated that both BAK and BAX were required for cell death induced by NOXA and ABT-263 treatment in HN12 cells (Fig 2C). BIM often plays an important role in cell death induced by combination treatments with ABT-263 [34]. However, knockdown of BIM did not affect cell death induced by NOXA (Fig 1D) and NOXA+ABT-263 combination (S1B Fig), suggesting that inhibition of anti-apoptotic BCL-2 family proteins are sufficient for direct activation of BAK/BAX. Although the levels of BCL-2, BIM, and NOXA were significantly different among the cell lines examined (S4 Fig), this variation did not explain why HN8, but not other cell lines, is sensitive to NOXA expression or fenretinide alone. Thus, further studies are needed to clarify.

**Fig 8:**
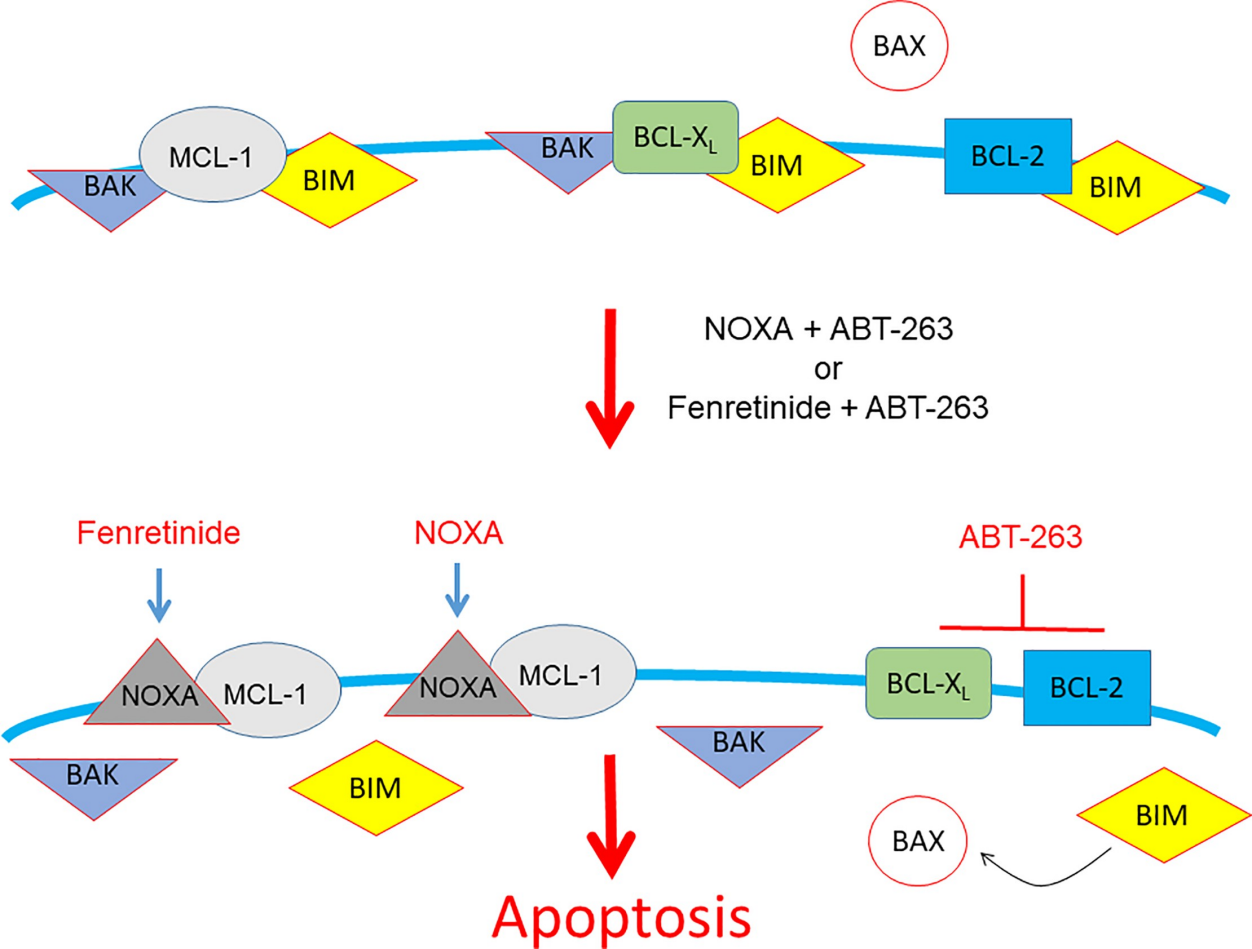
The mode of activity of agents used in this study. In untreated cells, pro-survival BCL-2, BCL-X_L_, and MCL-1 bind to inhibit the function of pro-apoptotic BAX and BAK. When cells are treated by combinations, these pro-apoptotic proteins are released and activated to efficiently induce apoptosis.

In addition to HN8 cells, other cell lines examined, regardless of the p53 or HPV statuses, exhibited an increase in apoptosis for the combination of NOXA or fenretinide with ABT-263, as compared to single treatments. These findings support the hypothesis that combination of NOXA or fenretinide with ABT-263 enhances cell death by interruption of BAK-MCL-1 interaction and functional inhibition of BCL-X_L_ in HNSCC cells (S3 Fig and Fig 8). A similar concept that co-inhibition of both BCL-X_L_ and MCL-1 is effective for HNSCC treatment has been published recently [35]. It has also been shown that a combination of fenretinide and ABT-737, a prototype of ABT-263, synergistically induces cell death in neuroblastoma [36]. Furthermore, NOXA induction by fenretinide is apparently independent of the p53 status and function (Figs 3A, 5C and 6A). These results indicate a couple of important clinical implications. It is well known that more than 50% of the HNSCC patients possess either p53 deletion or mutation [1, 2]. Furthermore, there is a significant increase of HPV-positive oropharyngeal carcinoma in recent years [37, 38]. Thus, fenretinide would be used as an alternative NOXA inducer in the context here, and combination of fenretinide and ABT-263 could directly activate the cell death machinery to overcome the status and function of p53.

ABT-263 is currently used in clinical trials for solid tumor treatments. However, there is dose-dependent thrombocytopenia as a side effect, because of BCL-X_L_-mediated survival of platelets [15, 39]. Combination of NOXA or fenretinide with the BCL-X_L_ specific inhibitor A-1331852, but not with the BCL-2 specific inhibitor ABT-199, showed similar amounts of cell death as compared to ABT-263 (Fig 7), strongly suggesting that the inhibition of MCL-1 and BCL-X_L_ is sufficient to induce cell death in HNSCC cells. It has been demonstrated that A-1331852 sufficiently recapitulates the efficacy of ABT-263 in combination with docetaxel for the treatment of solid tumors and less toxic in the mouse models [33]. Before entering in clinics, the strategy demonstrated here needs to be tested in the animal models of HNSCC such as patient-derived xenografts in immunodeficient mice and chemical carcinogen (e.g. 4-nitroquinoline 1-oxide)-induced HNSCC in immunocompetent mice [40].

## Supporting information

S1 Fig Neither BIM, BAK, nor BAX alone is sufficient to mediate cell death induced by NOXA + ABT-263 in HN12 cells. (A) HN12 cells were treated with Ad-Con (C), Ad-NOXA and ABT-263 (NA), Ad-Con with Q-VD-OPH (QVD+C), or Ad-NOXA and ABT-263 with QVD-OPH (QVD+NA). After 24 h, cells were analyzed using FACS (N = 3). Values represent the means ± S.D.
**Top:** Lentiviruses encoding short-hairpin BIM (shBIM), BAK, (shBAK), BAX (shBAX), and non-targeting control (shC) were infected in HN12 cells and stable cell lines were established with puromycin selection. Cells were then treated with Ad-Con (C) and Ad-NOXA (N) for 16 h followed by Western blot analyses. **Bottom:** The cells were treated with Ad-Con and Ad-NOXA for 24 h followed by FACS analyses to determine total amount of apoptosis (N = 3). Values represent the means ± S.D.
(TIF)

S2 Fig Evaluation of synergistic activity between fenretinide and ABT-263. Bliss independence analysis is shown after the treatment with fenretinide and ABT-263 across varying doses. Bliss scores greater than zero, close to zero, and less than zero represent synergy, additivity, and antagonism, respectively.
(TIF)

S3 Fig The interaction of BAK-MCL-1 and BAK-BCL-X_L_ with fenretinide + ABT-263 treatment. UMSCC1 cells were treated with fenretinide (10 μM) and ABT-263 (1 μM) for 16 h. Equal amounts of total extracts were incubated with anti-BAK antibodies followed by Western blots with the indicated antibodies. The input represents 20/500 of the immunoprecipitated lysates.
(TIF)

S4 Fig The expression of the BCL-2 family proteins in HNSCC cells lines used in this study. Equal amounts of the total extracts from each cell line were analyzed by Western blots with the indicated antibodies.
(TIF)
