# Supplementary figures and images for: Combination of fenretinide and ABT-263 induces apoptosis through NOXA for head and neck squamous cell carcinoma treatment
Source: PLoS One. 2019 Jul 5;14(7):e0219398. doi: 10.1371/journal.pone.0219398 (PMC6611623; doi:10.1371/journal.pone.0219398)

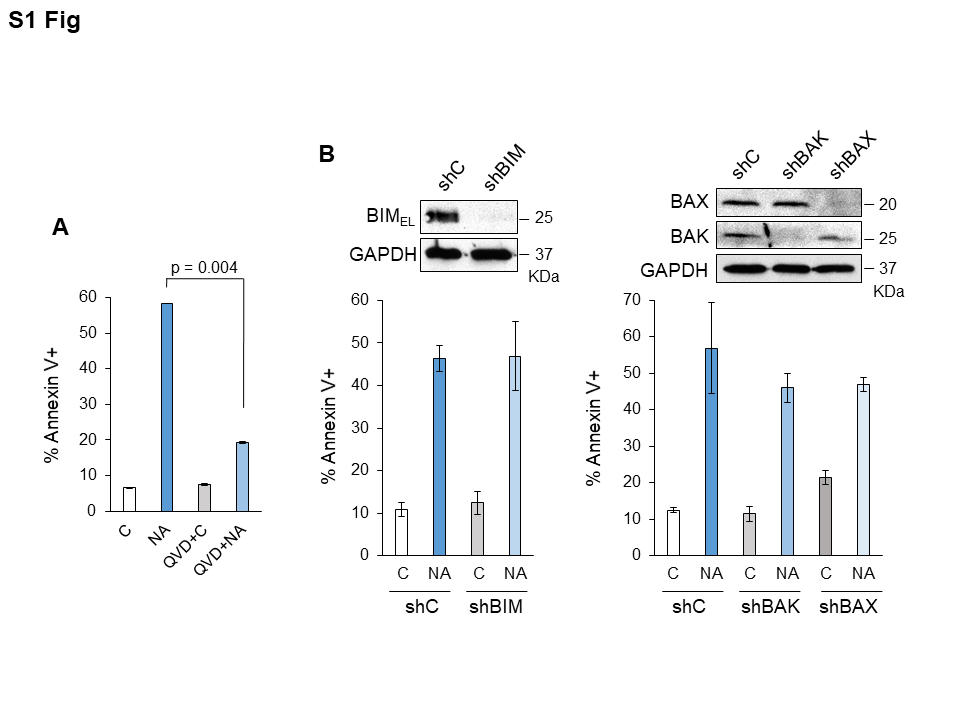

Supplement: S1 Fig — (A) HN12 cells were treated with Ad-Con (C), Ad-NOXA and ABT-263 (NA), Ad-Con with Q-VD-OPH (QVD+C), or Ad-NOXA and ABT-263 with QVD-OPH (QVD+NA). After 24 h, cells were analyzed using FACS (N = 3). Values represent the means ± S.D. Top: Lentiviruses encoding short-hairpin BIM (shBIM), BAK, (shBAK), BAX (shBAX), and non-targeting control (shC) were infected in HN12 cells and stable cell lines were established with puromycin selection. Cells were then treated with Ad-Con (C) and Ad-NOXA (N) for 16 h followed by Western blot analyses. Bottom: The cells were treated with Ad-Con and Ad-NOXA for 24 h followed by FACS analyses to determine total amount of apoptosis (N = 3). Values represent the means ± S.D. (TIF) [file pone.0219398.s001.TIF]

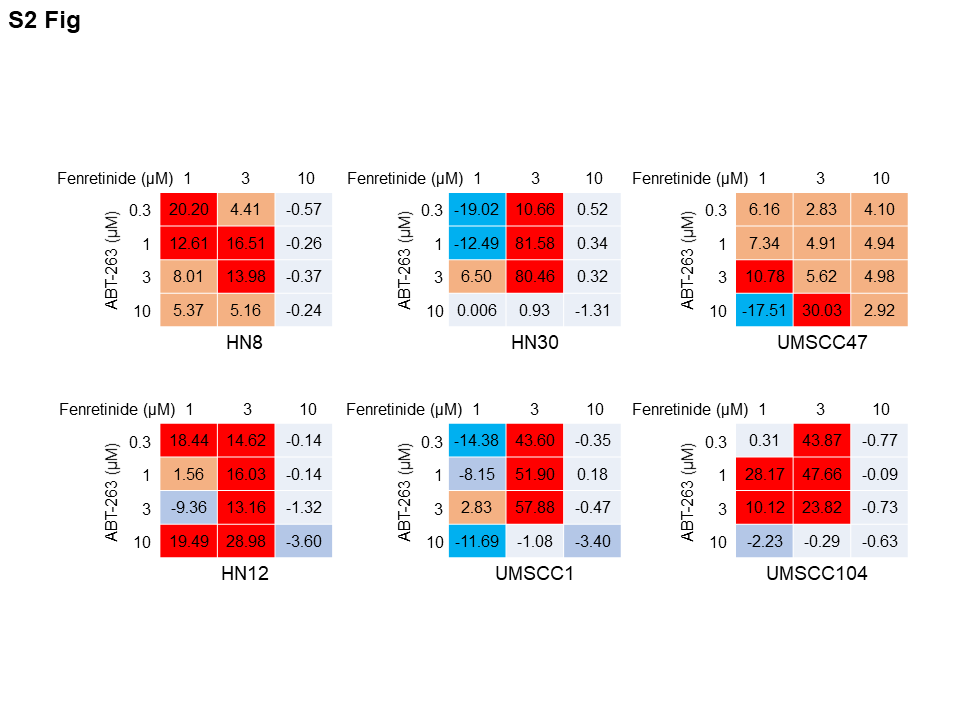

Supplement: S2 Fig — Bliss independence analysis is shown after the treatment with fenretinide and ABT-263 across varying doses. Bliss scores greater than zero, close to zero, and less than zero represent synergy, additivity, and antagonism, respectively. (TIF) [file pone.0219398.s002.TIF]

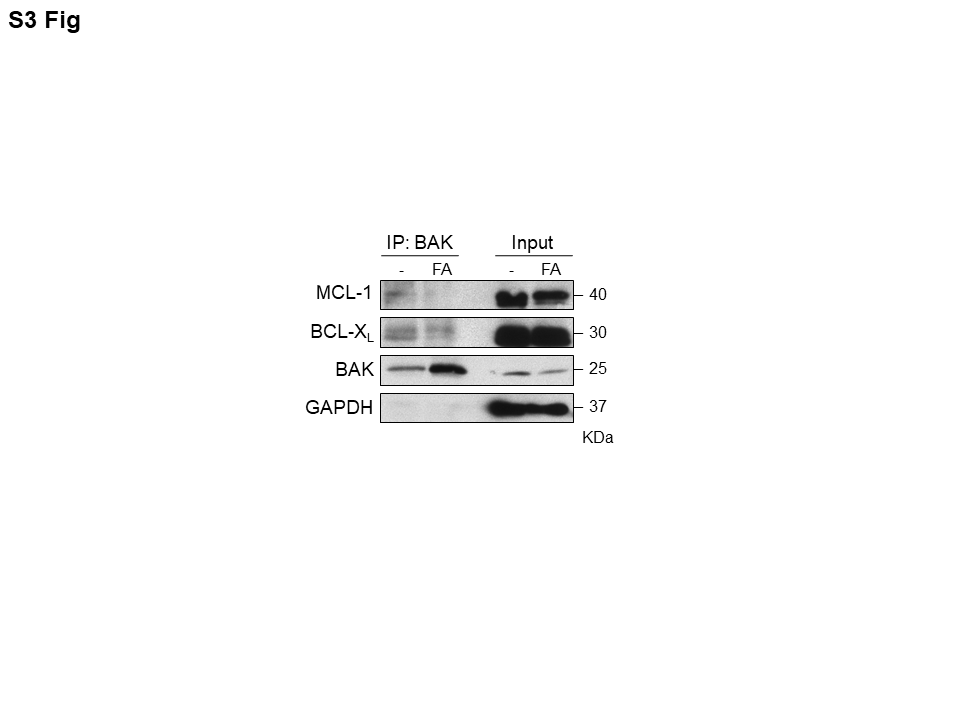

Supplement: S3 Fig — UMSCC1 cells were treated with fenretinide (10 μM) and ABT-263 (1 μM) for 16 h. Equal amounts of total extracts were incubated with anti-BAK antibodies followed by Western blots with the indicated antibodies. The input represents 20/500 of the immunoprecipitated lysates. (TIF) [file pone.0219398.s003.TIF]

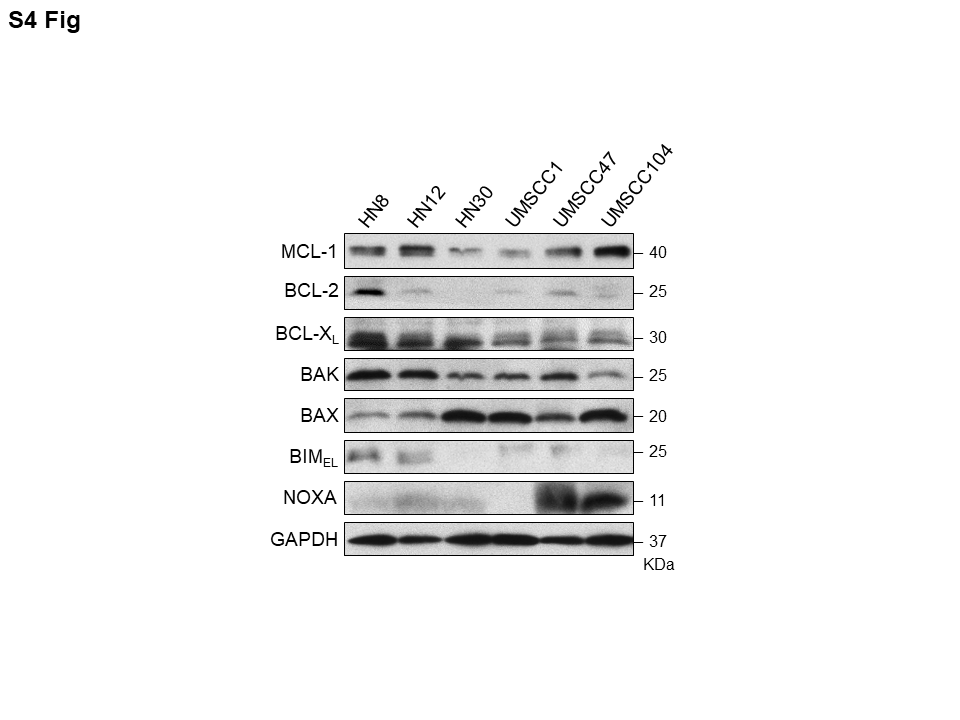

Supplement: S4 Fig — Equal amounts of the total extracts from each cell line were analyzed by Western blots with the indicated antibodies. (TIF) [file pone.0219398.s004.TIF]
